# Development and characterization of an immortalized swine respiratory cell line for influenza A virus research

**DOI:** 10.3389/fvets.2023.1258269

**Published:** 2023-12-18

**Authors:** Peter J. Neasham, Vasilis C. Pliasas, J. Fletcher North, Celeste Johnson, S. Mark Tompkins, Constantinos S. Kyriakis

**Affiliations:** ^1^Department of Pathobiology, College of Veterinary Medicine, Auburn University, Auburn, AL, United States; ^2^Emory-UGA Center of Excellence for Influenza Research and Surveillance (CEIRS), Atlanta, GA, United States; ^3^Center for Vaccines and Immunology, University of Georgia, Athens, GA, United States

**Keywords:** swine, influenza A virus, primary respiratory cells, immortalized respiratory cells, cells characterization

## Abstract

**Introduction:**

Swine serve as an important intermediate host species for generating novel influenza A viruses (IAVs) with pandemic potential because of the host’s susceptibility to IAVs of swine, human and avian origin. Primary respiratory cell lines are used in IAV research to model the host’s upper respiratory tract *in vitro*. However, primary cell lines are limited by their passaging capacity and are time-consuming for use in industry and research pipelines. We were interested in developing and characterizing a biologically relevant immortalized swine respiratory cell line that could be used for efficient propagation and characterization of swine IAV isolates.

**Methods:**

Lung tissue for the generation of primary swine respiratory cells were isolated from the bronchi of an 8-week-old Yorkshire/Hampshire pig, which were immortalized by transduction of the SV40 T antigen using a lentivirus vector. The transduction of the SV40 T antigen was confirmed by Real Time RT-PCR in cells passaged greater than twenty times.

**Results:**

Immortalized swine respiratory cells expressed primarily α2,6 sialic acid receptors and were susceptible to both swine and human IAVs, with swine viruses exhibiting higher replication rates. Notably, infection with a swine H3N2 isolate prompted increased IL-6 and IL-1α protein secretion compared to a seasonal human H3N2 virus. Even after 20 passages, the immortalized cells maintained the primary respiratory cell phenotype and remained permissive to IAV infection without exogenous trypsin.

**Discussion:**

In summary, our developed immortalized swine respiratory cell line offers an alternative *in vitro* substrate for studying IAV replication and transmission dynamics in pigs, overcoming the limitations of primary respiratory cells in terms of low passage survivability and cost.

## Introduction

Influenza A viruses (IAV) are enveloped, negative sensed RNA viruses with a single stranded and segmented genome, which belong to the *Orthomyxoviridae* family. Wild birds serve as the natural host reservoir for IAV. However, IAV has a relatively wide host range and has been isolated in both avian and mammalian species including: domesticated birds, humans, swine, canines, equines, felines, and aquatic mammals. The ability of IAV to jump multiple species barriers is enabled by the segmented nature of the IAVs genome, which enables reassortment of whole gene segments between co-infecting IAV strains and lack of a proofreading mechanism enabling rapid drift of the viruses surface glycoproteins (1).

In mammalian hosts, IAV is known to cause repiratory disease. IAV enters into respiratory tract and enters into primariliy respiratory epithelial cells that express glycoprotein receptors that terminate with sialic acids (SA). The SA is attached to a galactose molecule, which provides a recognition site for the receptor binding domain (RBS) of the IAV haemagluttin (HA) protein, facilitating receptor mediated endocytosis. The HA protein can bind to SA recptors in either an 2,3 or 2,6 linked confirmation. Strains of avian-origin preferentially bind to host cells in an 2,3 confirguration, whilst mammalian-origin strains preferentially bind in an 2,6 configuration (2–6). The distribution of SA receptors is implicated as a host range factor that IAVs must overcome to cross species barriers.

In the human and swine hosts, the distribution of α2,3 and α2,6 linked SA are similar in the respiratory tract (7–9). However, IAVs encoding solely human (hu) origin gene segment are isolated from swine during passive survellance (10–13).This suggests that reverse zoonotic transmission of IAVs at the human/swine interface is not solely dependent on SA distribution of the host, and multiple host and viral factors are involved with the ability of the IAV to efficiently transmit and replicate within the swine host (14).

Cell lines are often used in IAV research to propagate and to assess the transmission and replicationary potential of novel isolates within a particular host for pandemic prepardation. Primary respiratory epithelial cell lines are currently the gold-standard cellular model for assessing the physical properties of IAV strains and can be fully differentiated in an air–liquid interface (ALI) system to produce a pseudostratified epithelium, containing ciliated cells and goblet cells, that models the respiratory structure and architechture of the hosts lungs *in vitro* (3, 15–18). However, primary cell lines are limited by the low number of passages before reaching senescense, susceptibility to contamination and verification of the absense of pathogens, high cost of reagents needed to grow and maintain the cells in ALI, donor-to-donor varibility, availability of host tissue, and the time taken to fully differentiate the cells (3–4 weeks in ALI). Consequently, immortalized respiratory cell lines are used as an alternative cellular model to study the properties of IAV isolates to a susceptible host. However, immortalized respiratory cell lines may have altered physical properties to the primary host cell. This includes losing the ability to secrete proteases neccesary for IAV entry into the host cell and differentiate. Immortalized swine tracheal (siTEC) and nasal epithelial (siNEC) cells have been previously developed by transducing the SV40-T antigen using a lentivirus vector (19). The siTEC and siNEC cell lines were permissible to H1N1, H1N2, and H3N2 IAV strains and retained the functional characteristics of the primary cells (19).

In this study, we developed an immortalized swine bronchial epithelial cell line for IAV research by introducing Simian Virus 40 (SV40-T) antigen into primary swine respiratory cells harvested from the bronchi of an 8 weeks old porcine reproductive and respiratory virus (PRRSv) and IAV seronegative pig. Porcine bronchial epithelial cells (PBEC) have been immortalized by delivering human telomerase reverse transcriptase (h-TERT), to extend the replicative capacity of cells through telomerase extention (20). However, our primary objective was to establish an immortalized swine bronchial epithelial cell line via transduction of SV40-T antigen.

The immortalized swine respiratory cells appeared mostly of epithelial origin and retained morphological characteristics of the swine primary cells. In addition, both primary and immortalized swine respiratory cells were permissive to human (huIAV) and swine (swIAV) IAVs of H1N1, H1N2, and H3N2 subtypes that most predominantly circulate at the human/swine interface and modelled host restriction of wholly human-origin H3N2 observed in the swine host. Our results suggest that the immortalized swine respiratory cells could be used as an appropriate immortalized cellular model for the lower respiratory tract of the swine host and may serve as a tool to study the transmission and replicationary potential of novel IAV strains.

## Materials and methods

### Cell culture and virus stocks

Madin-Darby canine kidney (MDCK) cells were cultured in Dulbecco’s modified Eagles’ medium (DMEM) (Invitrogen), supplemented with 10% fetal bovine serum (FBS) and 1% penicillin–streptomycin. The MDCK cells were maintained at 37°C in a 5% CO2 atmosphere. Influenza A viruses used in this study were propogated in MDCK cells (ATCC). Virus titers were quantified by TCID_50_ and calculated using the Reed and Muench method (21).

### Harvesting, isolation, and immortalization of primary respiratory swine cells

Lung tissue for the isolation of swine respiratory cells were collected from an 8 weeks-old Yorkshire/Hampshire pig. Lung tissue was rinsed in phosphate-buffered saline (PBS) and sections from the bronchi were sliced into small pieces. The sliced bronchial tissue was incubated for 2 h with 800 U collagenase at 37°C and 5% CO2. Following collagenase digestion, respiratory cells were strained through a 70 μm cell strainer and centrifuged. The cell pellet was washed twice with PBS and cells were seeded onto collagen coated flasks. Cells were incubated for 24 h at 37°C and 5% CO2 in DMEM/F12 supplemented with FBS, retinoic acid, bovine pituitary extract (BPE), epidermal growth factor, cholera toxin, transferrin, insulin, and penicillin/streptomycin (growth media). Following the 24 h incubation, non-adherent cells were transferred to rat-tail collagen (Corning) coated flask and cultured until fully confluent.

At passage four, primary swine respiratory cells were plated in growth media onto rat-tail collagen coated 6-well plates (1 × 10^5^). Once the cells reached 70% confluence, the growth media was removed, and cells were washed with PBS. Target primary swine respiratory cells were infected and incubated overnight at 37°C and 5% CO2 with 10^7^ Lenti-SV40T vector (abm) in the presence of 10 μg/mL polybrene. Following the overnight incubation, supernatant from lentivirus infected swine cells was removed, and cells were washed with PBS. Fresh growth media was added to the cells, which were incubated for 72 h at 37°C and 5% CO2_._ Immortalized swine respiratory cells were passaged >20 times and successful transduction of the SV40 T antigen gene into the immortalized swine respiratory cells was confirmed by quantitative reverse transcription polymerase chain reaction (RT-qPCR) using SV40 T antigen targeting primers 5’ ACTGAGGGGCCTGAAATGA, 5’ GACTCAGGGCATGAAAC AGG. Swine respiratory cells were confirmed of swine origin using swine GAPDH targeting primers 5’ ACCCAGAAGACTGTGGATGG and 5’ ACGCCTGCTTCACCACCTTC.

### Immunoflurescent staining and confocal microscopy

Immortalized (P20) and primary (P6) swine respiratory cells were seeded (1 × 10^5^) into 6-well plates to 70–80% confluency onto coverslips coated with rat-tail collagen (Corning) and inoculated with either control/PBS, A/TX/12/H3N2 or A/swine/MN/12/H3N2 at an MOI of 0.1 diluted in PBS (−/−). At 24 hpi, cells were washed twice and fixed using 4% paraformaldehyde and permeablised with 0.5% TritonX/PBS for 30 min. The cells were blocked in 10% goat serum with PBS/Tween20 for 1 h prior to staining with the primary antibodies:

### Cytokeratin 18 staining

Immortalized and primary swine respiratory cells were incubated for 1 h with anti-cytokeratin 18 mouse monoclonal antibody (Abcam) (1,200).

### Vimentin staining

Immortalized and primary swine respiratory cells were incubated for 1 h with anti-vimentin rabbit monoclonal antibody (Abcam) (1:400).

### Lectin staining

Immortalized and primary swine respiratory cells were incubated for 1 h with biotinylated *Maakia amurensis* lectin I and II (Vector^®^ Laboratories) (1:800), to stain α2,3-linked SA receptors or fluorescein isothiocyanate (FITC)-conjugated *Sambucus nigra agglutinin* (Vector^®^ Laboratories) (1,200) to stain for α2,6-linked SA receptors.

### Influenza A virus staining

Immortalized swine respiratory cells were incubated for 45 min with anti-alpha tubulin (1:800), anti-beta-tubulin (1:200), and anti-influenza A nucleoprotein (1:500).

After staining with the apprapriate primary antibodies, cells were washed 3× with PBS/Tween20 and stained with secondary antibodies: goat anti-mouse Alexa Fluor 546 (Invitrogen) (1:400), goat anti-rabbit Alexa Fluor 488 (Invitrogen) (1:400) or goat anti-mouse FITC (1:400). Coverslips were transferred to slides and mounted using slowfade mounting medium (Invitrogen). The slides were visualized using ECHO revolve flourescent microscope (ECHO A Bico Company, San Diego, CA). All captured images were edited and process using FIJI software (22).

### Influenza A virus infections

Immortalized (P24) swine respiratory cells were seeded (1 × 10^5^) onto 6-well plates and cultured in growth media onto rat-tail collegen (Corning) coated 6-well plates until 80% confluency. Cells were then washed in PBS and inoculated with a panel of hu- and swIAVs (Table 1) at an MOI of 0.1 diluted in PBS (−/−). Following IAV inoculation, cells were incubated for 2 h at 37°C and 5% CO2 before washing cells with PBS and replacing the inoculum with fresh FBS free growth media. The inoculated cells were incubated at 37°C and 5% CO2 until the appropriate end point of collection (0-, 24-, 48-, 72-hpi). At this point, cell supernatant was harvested and IAV was quantified by TCID_50_ and calculated using the Reed and Muench method as previously described. Samples were analyzed using three biological replicates from each time point.

**Table 1:** Immortalized swine respiratory cells are permissible to hu and swIAV strains of H1N1, H1N2, and H3N2 subtypes.

Virus name	Abbreviation	Subtype	Origin	Virus Titer 24 hpi	Virus Titer 48 hpi	Virus Titer 72 hpi	Area under curve
				(log10 TCID50/mL)	(log10 TCID50/mL)	(log10 TCID50/mL)	
A/Puerto Rico/8/1934	A/PR8/1935	H1N1	Human	4.88 ± 0.22	6.21 ± 0.29	5.22 ± 0.11	329 ± 11.09
A/New Caledonia/20/99	A/NewCal/1999	H1N1	Human	4.77 ± 0.27	6.11 ± 0.11	5.55 ± 0.22	327.9 ± 9.90
A/Brisbane/10/2007	A/Bris/2007	H3N2	Human	3.83 ± 0.16	5.22 ± 0.11	4.61 ± 0.05	272.6 ± 5.97
A/Perth/16/2009	A/Perth/2009	H1N1	Human	2.38 ± 0.39	3.89 ± 0.11	2.78 ± 0.27	183.9 ± 13.27
A/CA/07/2009	A/CA/2009	H1N1	Human/pdm	4.77 ± 0.11	6.05 ± 0.27	6.5 ± 0.5	337.8 ± 13.61
A/Victoria/361/2011	A/VIC/2011	H3N2	Human	4.55 ± 0.05	5.33 ± 0.16	3.77 ± 0.39	282.6 ± 9.77
A/TX/50/2012	A/TX/2012	H3N2	Human	3.6 ± 0.05	4.6 ± 0.05	1.17 ± 0.16	211.1 ± 4.11
A/swine/MN/A01125993/2012	A/swine/MN/2012	H3N2	Swine	7.34 ± 0.05	7.44 ± 0.11	6.89 ± 0.11	438.5 ± 4.33
A/Hong Kong/4081/2014	A/HK/2014	H3N2	Human	3.11 ± 0.11	3.88 ± 0.22	1.67 ± 0.16	181.8 ± 8.09
A/swine/NC/KH1552516/2016	A/swine/NC/2016	H3N2	Swine	3.55 ± 0.46	6.28 ± 0.14	7.05 ± 0.24	320.5 ± 15.74
A/WI/588/2019	A/WI/2019	H1N1	Human/pdm	4.6 ± 0.05	6.05 ± 0.27	5.94 ± 0.24	327.2 ± 9.68
A/swine/GA/27480/2019	A/swine/GA/2019	H1N2	Swine	5.55 ± 0.11	7.55 ± 0.22	7.83 ± 0.16	408.5 ± 8.09

Replication kinetics of H1N1, H1N2, and H3N2 hu and swIAV subtypes in immortalized swine respiratory cells. Immortalized swine respiratory cells were seeded onto rat-tail collagen coated 6-well and inoculated independently with H1N1 or H3N2 huIAVs, or H1N2, or H3N2 swIAV strains at an MOI of 0.1. Supernatant was harvested at 0-, 24-, 48-, and 72 h post infection and IAV titers were measured by TCID_50_. The averages of three replicates. ± represents standard error (*n* = 3).

### Quantification of cytokine/chemokine secretion from primary and immortalized swine respiratory cells

Supernatant from primary (P6) and immortalized (P24) swine respiratory cell inoculated with either A/TX/12/H3N2 or A/swine/MN/12/H3N2 at an MOI of 0.1 harvested at −24 and −48 hpi was analyzed for IL-1α, IL-6, and IL-8 secretion using the MILLIPLEX MAP Porcine Cytokine/Chemokine Magnetic Bead panel per manufacture’s instruction (EMD Millipore Corporation, Billerica, MA).

### Statistics

Statistical analysis was performed using GraphPad Prism 9.3.1. A two-way ANOVA was used to identify significance differences between IL-1α, IL-6, and IL-8 secreted from the immortalized (P24) and primary (P6) swine respiratory cells that were either mock infected (PBS), or inoculated independently with A/TX/12/H3N2 or A/swine/MN/12/H3N2. To determine the percentage of vimentin positive cells in the immortalized (P20) and primary (P6) swine respiratory cell lines, IF images taken on the ECHO revolve flourescent microscope (ECHO A Bico Company, San Diego, CA) were processed using FIJI software and vimentin positive cells were counted and subtracted from the number of DAPI positive cells (*n* = 3).

## Results

### Immortalized swine respiratory cells maintain functional properties of primary cell counterparts

**Figure 1:**
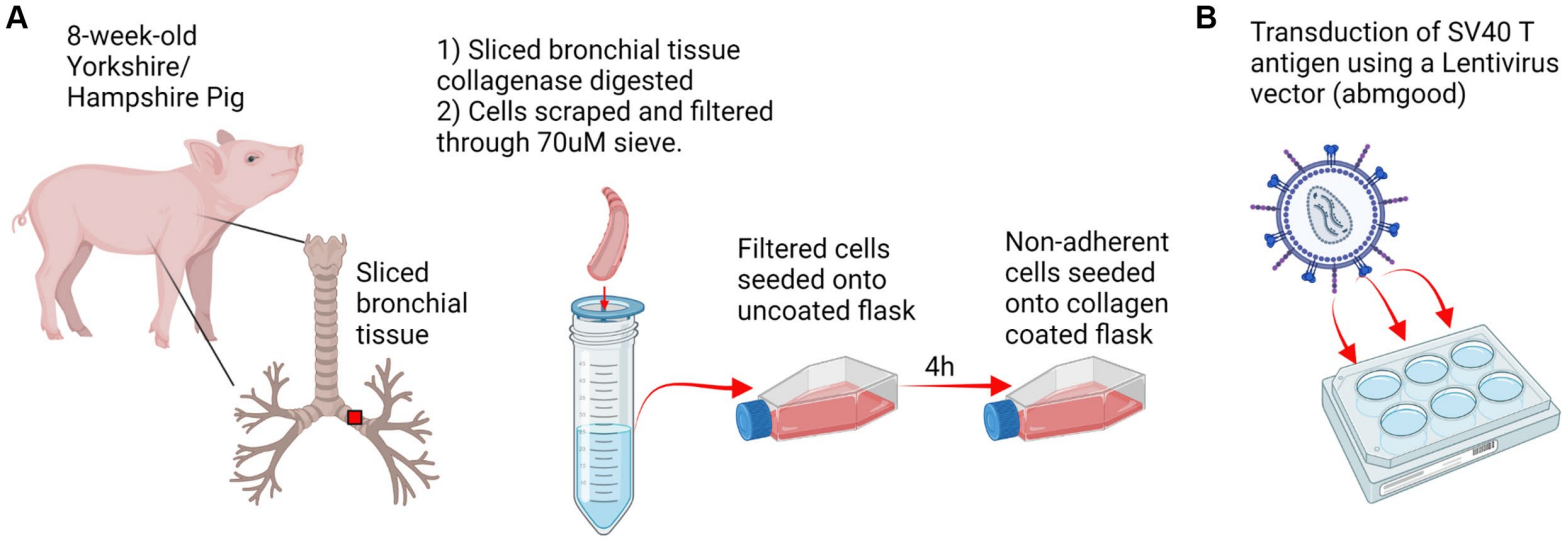
Experimental design. **(A)** Respiratory tract tissue was harvested from an 8 weeks-old Yorkshire/Hampshire pig. 1 cm bronchial tissue slices were collagenase digested for 2 h at 37°C and swine respiratory cells were scraped and filtered through a 70 μM sieve. Primary swine respiratory cells were seeded onto uncoated flasks and non-adherent cells were transferred to a collagen coated flask following 4 h incubation. **(B)** Transduction of SV40 T antigen. Primary swine respiratory cells were seeded onto 6-well plates and inoculated with 10^7 SV40-lentivirus in the presence of polybrene. Positive transduction and retention of the SV40 T antigen was confirmed by RT-PCR at passage 20. Created with BioRender.com.

**Figure 2:**
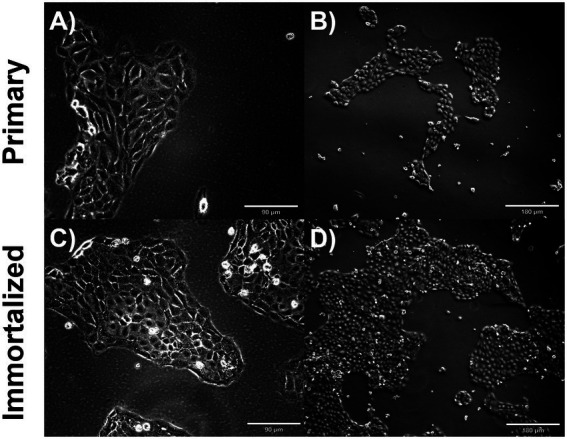
Immortalized and primary swine respiratory cell phenotype. Immortalized and primary swine respiratory cells were seeded onto collagen coated flasks and incubated at 37°C and 5% CO2 for 2 days. Bright field primary respiratory cells at P6 **(A)** Magnification ×10, Scale Bar 90 μm, **(B)** Magnification ×20, Scale Bar 180 μm. Bright field primary swine respiratory cells at P20 **(C)** Magnification ×10, Scale Bar 90 μm **(D)** Magnification ×20, Scale Bar 180 μm.

**Figure 3:**
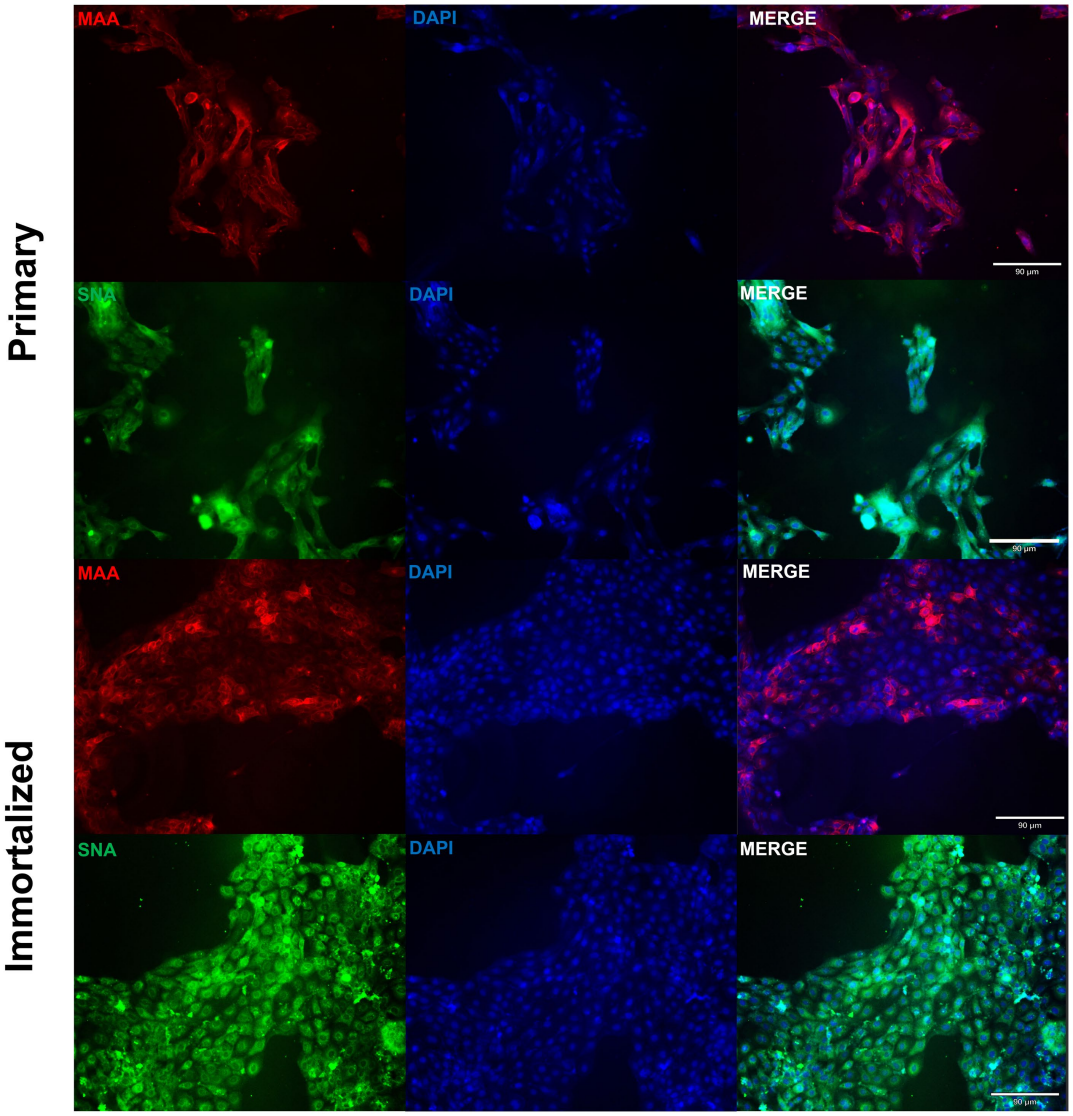
Primary and immortalized swine respiratory cells express α2,6 sialic acid receptors. Primary (P6) and immortalized swine respiratory (P20) cells were seeded onto 6-well rat-tail collagen coated coverslips and stained with DAPI (blue) and either *Maackia amurensis* (MAA, red) or *Sambucus nigra agglutinin* (SNA, green) lectins. Magnification ×10, Scale Bar 90 μm.

**Figure 4:**
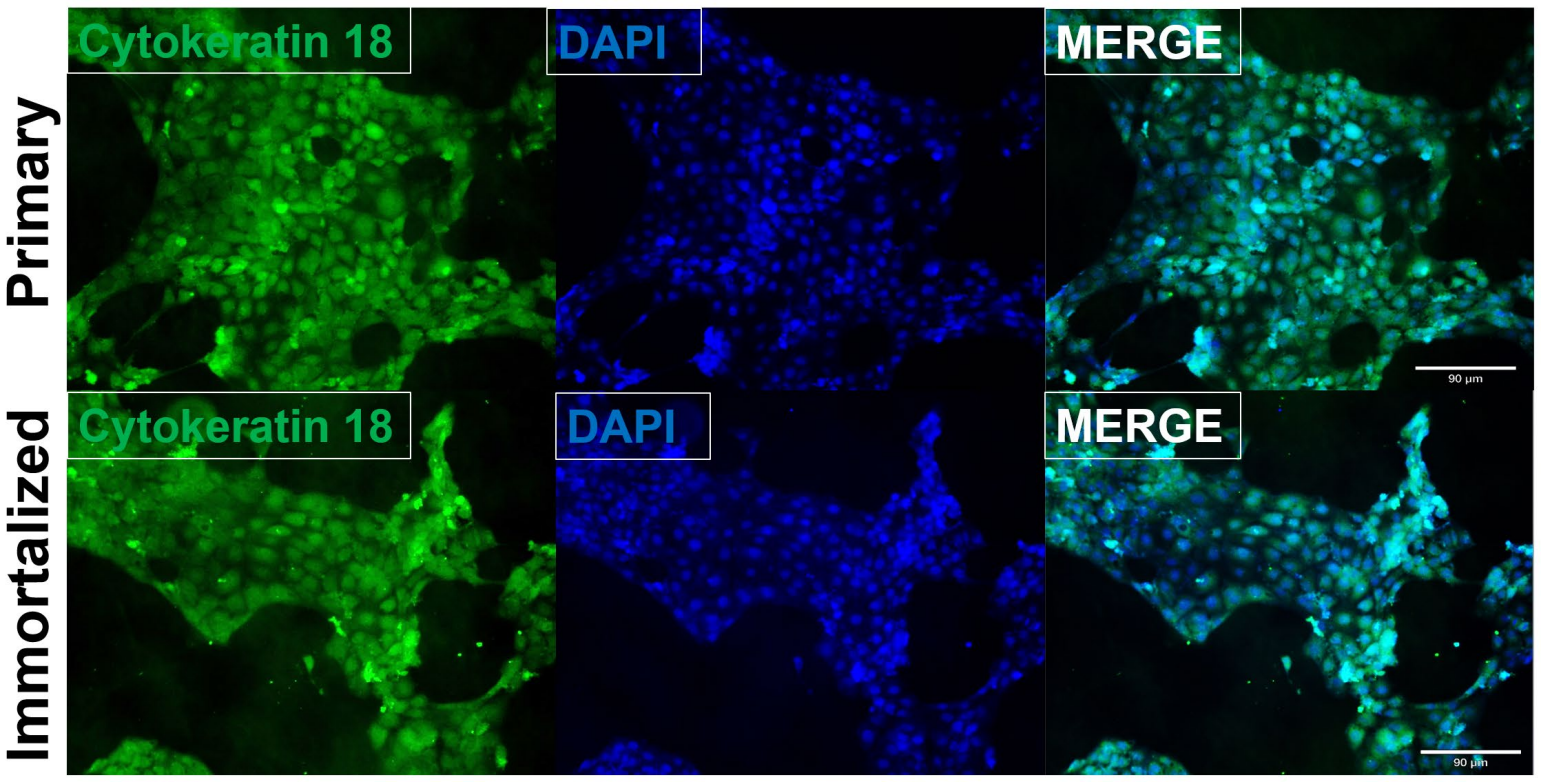
Immortalized swine respiratory cells are primarily of epithelial origin. Following harvesting, isolation, and immortalization, both the primary (P6) and immortalized (P20) swine respiratory cells were seeded onto 6 well rat-tail collagen coated coverslips and stained for cytokeratin 18 (green) and DAPI (blue). Magnification ×10.

Primary swine respiratory cells were isolated from the bronchi of an 8 weeks old Yorkshire/Hampshire and immortalized by transduction of the SV40-T antigen following 4 successful passages (Figure 1). The immortalized swine respiratory cells were successfully passaged >20 times, whilst primary swine respiratory cells failed to proliferate at P16. SV40 T antigen retention in the immortalized swine respiratory cells was confirmed by rt-qPCR, with a mean SV40-T antigen Cq value of 27.7 (*n* = 3) in cells passaged 20 times (Supplementary Table S1). Visually, immortalized swine respiratory cells maintained the morphological features of primary swine respiratory epithelial cells, such as, possessing the ability to form tight-junctions and a cobble-stone like appearance (Figure 2). The primary and immortalized swine respiratory cells expressed both α2,3 and α2,6 linked SA receptors, which are an important host restriction factor for IAV entry into the host cell (Figure 3). Both primary and immortalized cell lines expressed α2,6 linked SA receptors at a higher abundance compared to α2,3 linked SA receptors (Figure 3). In addition, the immortalized swine respiratory cells appeared to be mostly of epithelial origin, which was demonstrated by the majority of visualized cells expressing the epithelial marker cytokeratin 18 (Figure 4). Staining with the epithelial-mesenchymal transition and fibroblast marker vimentin, demonstrated that the isolated primary swine respiratory cells contained a high percentage (56.7%) of contaminating cells (Supplementary Figure S1). However, following trypsin-treatment and immortalization via SV40-T antigen transduction, we observed a decrease in the mean percentage of vimentin positive cells (32.08%), which was maintained up to 20 passages (Supplementary Figure S1).

**Figure 5:**
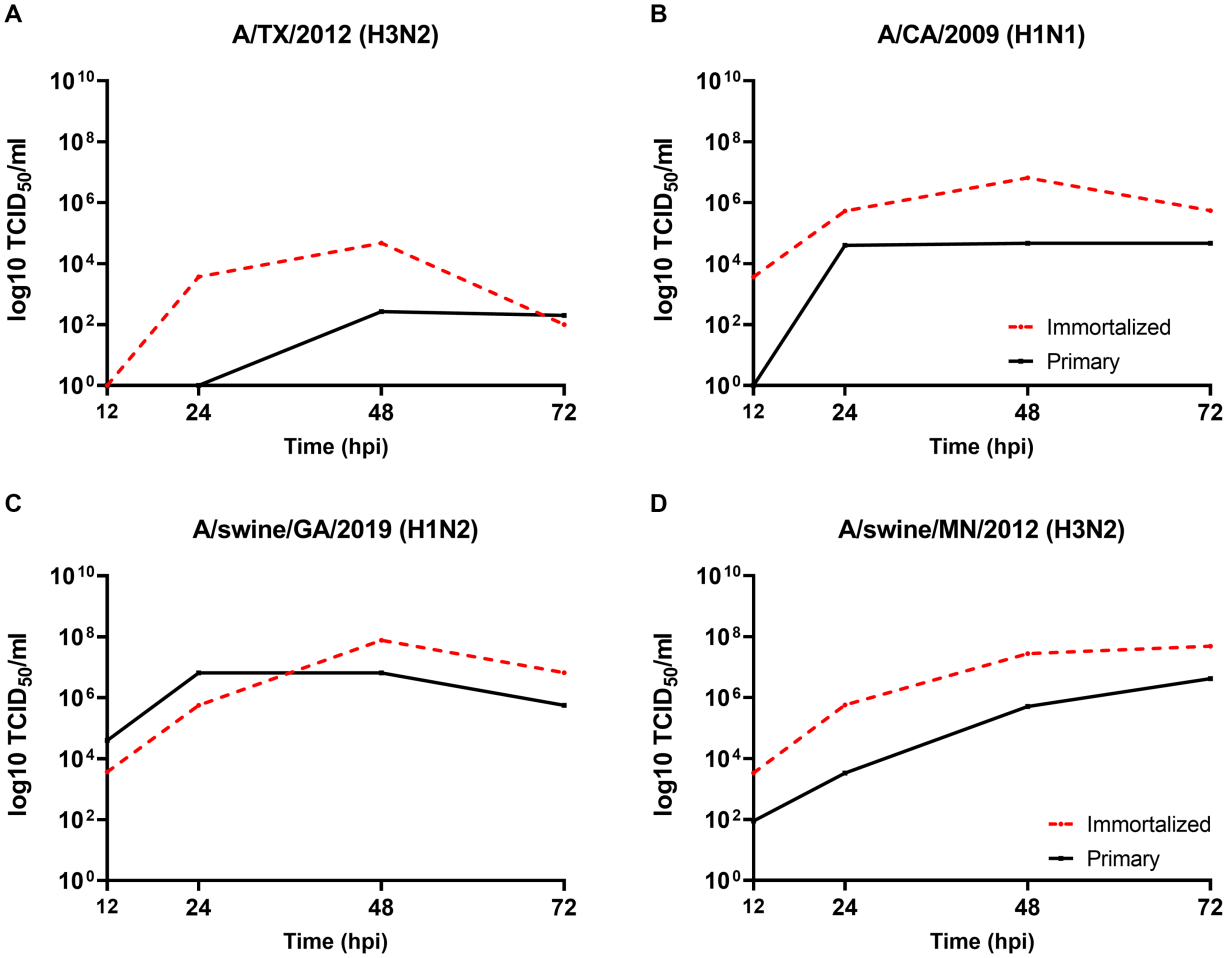
Primary and immortalized swine respiratory cells are permissible to hu- and swIAVs. Primary (solid black line) and Immortalized (dashed red line) respiratory swine cells (P6 and P24 respectively), were seeded onto 6-well rat-tail collagen coated coverslips and inoculated independently with either A/TX/2012/H3N2 **(A)**, A/CA/2009/H1N1 **(B)**, A/swine/GA/2019/H1N2 **(C)**, or A/swine/MN/2012/H3N2 **(D)** at an MOI of 0.1. Supernatant was harvest, 12-, 24-, 48-, and 72-hpi and viral titers were quantified by TCID_50_.

We evaluated the permissibility of either cell lines to IAV’s of mammalian origin, given the similar distribution of SA receptors to the swine hosts respiratory tract. Both primary and immortalized swine respiratory cell lines were permissive to A/TX/2012/H3N2 (Figure 5A), pdm A/CA/2009/H1N1 (Figure 5B) huIAV strains, A/swine/GA/2019/H1N2 (Figure 5C), and A/swine/MN/2012/H3N2 swIAV strains (Figure 5D). However, A/TX/2012/H3N2 replicated to lower titers compared to the other panel of IAV strains, which was most pronounced at 72-hpi (Figure 5A). This trend was reflected in both the primary and immortalized swine respiratory cell lines, which suggests that the host specificity of swine respiratory cells was not impacted by immortalization via SV40 T antigen transduction.

### Immortalized swine respiratory cells are permissible to IAV strains of human and swine H1N1, H1N2, and H3N2 subtypes

The viral replication kinetics of both human and swine-origin strains were evaluated to assess the permissiveness of the immortalized cells to IAV strains of H1N1, H1N2, and H3N2 subtypes. The panel of strains were selected to represent IAV subtypes that commonly circulate at the human/swine interface. The immortalized swine respiratory cells were permissible to the entire panel of IAV strains, regardless of IAV host-origin or subtype (Table 1). However, we did measure differences in the ability of individual strains to replicate within the immortalized cell line. Generally, huIAV H3N2 strains replicated poorly within the immortalized swine respiratory cell compared to swIAVs. The highest difference between peak titers were measured between A/swine/GA/2019/H1N2 (7.83 TCID_50/_mL), and A/TX/2012/H3N2 (1.17 TCID_50_/mL) at 72-hpi. Whilst the greatest difference in area under curve (AUC) was observed between A/swine/MN/2012/H3N2 (438.5), and the A/HongKong/2014/H3N2 huIAV strain (181.8). Overall, the replication kinetics of the panel of IAVs imply that the swIAVs replicate more efficiently in the immortalized swine respiratory cells, which provides evidence of swine host specificity.

We observed no differences in the viral titers and AUC values between A/CA/2009/H1N1 (pdm strain), A/NewCal/1999/H1N1 (pre-pandemic strain), and A/WI/2019/H1N1 (post-pandemic-like strain) at 24-, 48-, and 72-hpi (Table 1).

### Wholly huIAV H3N2 strains replicate poorly and produce a mild pro-inflammatory cytokine response in immortalized swine respiratory cells

**Figure 6:**
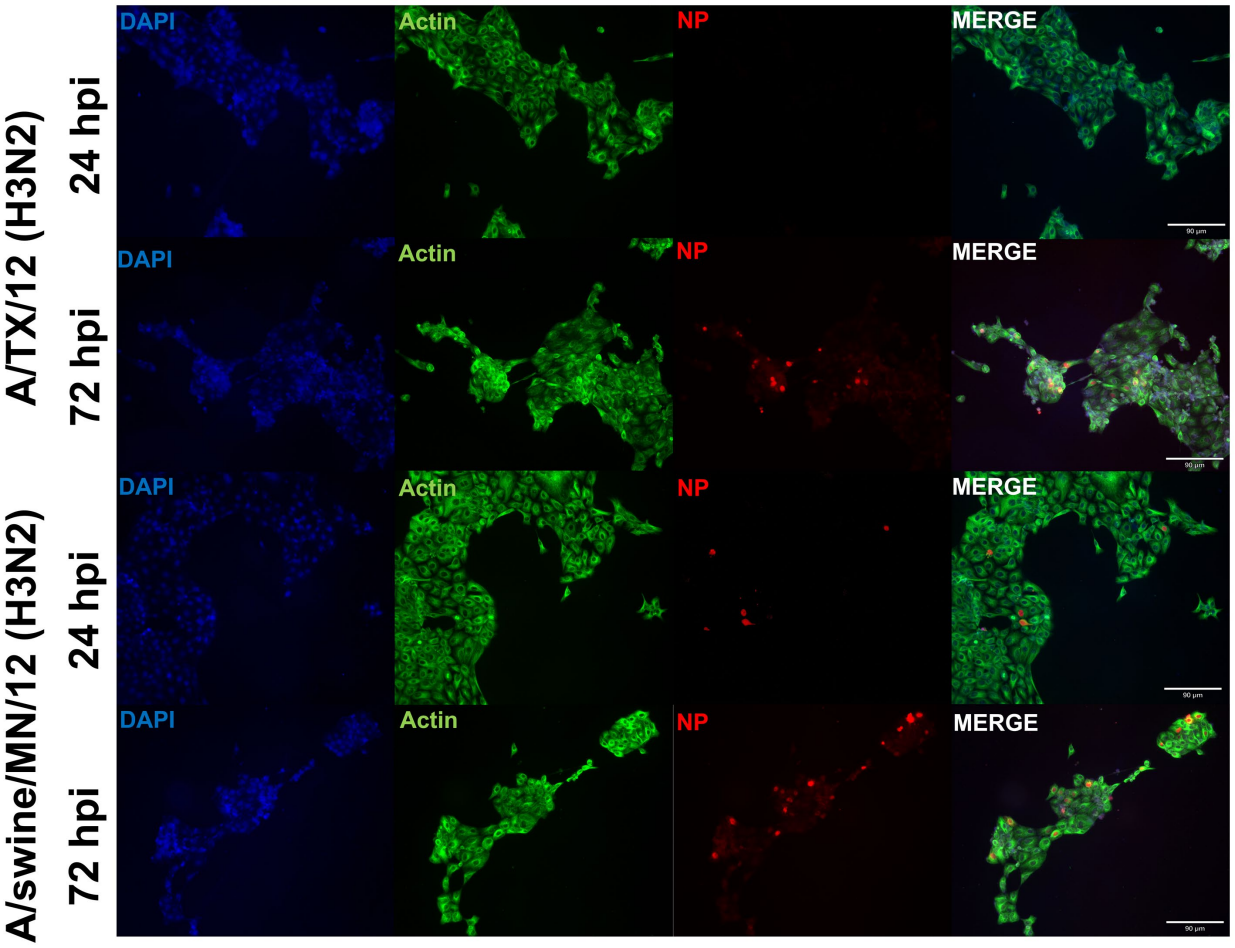
Human-origin H3N2 strain replicates poorly in immortalized swine respiratory cells compared to swIAV H3N2 counterpart. Immortalized swine respiratory cells (P20) were seeded onto 6-well rat-tail collagen coated coverslips and inoculated independently with either A/swine/MN/12/H3N2 or A/TX/2012.H3N2 at an MOI of 0.1. At 24 hpi, coverslips were stained with NP (red), alpha/beta actin (green), and DAPI (blue). Magnification ×10, Scale Bar 90 μm.

From the panel of H1N1, H1N2, and H3N2 hu and sw-origin IAVs evaluated in the immortalized swine respiratory cells (Table 1), we selected A/TX/2012/H3N2 and A/swine/MN/2012/H3N2 strain, encoding the triple reassortment internal gene (TRIG) cassette, to compare the replication and pro-inflammatory responses between wholly huIAV and swIAV H3N2 strains. Using immunohistochemistry, we were unable to visualize A/TX/2012/H3N2 nucleoprotein (NP) at 24-hpi (Figure 6). Although, both the A/TX/2012/H3N2 and A/swine/MN/2012/H3N2 NP protein was visualized at 72-hpi (Figure 6). This showed that there was a delay in the replication of the wholly huIAV H3N2 strain, compared to the TRIG swIAV H3N2 strain in the immortalized swine respiratory cells.

**Figure 7:**
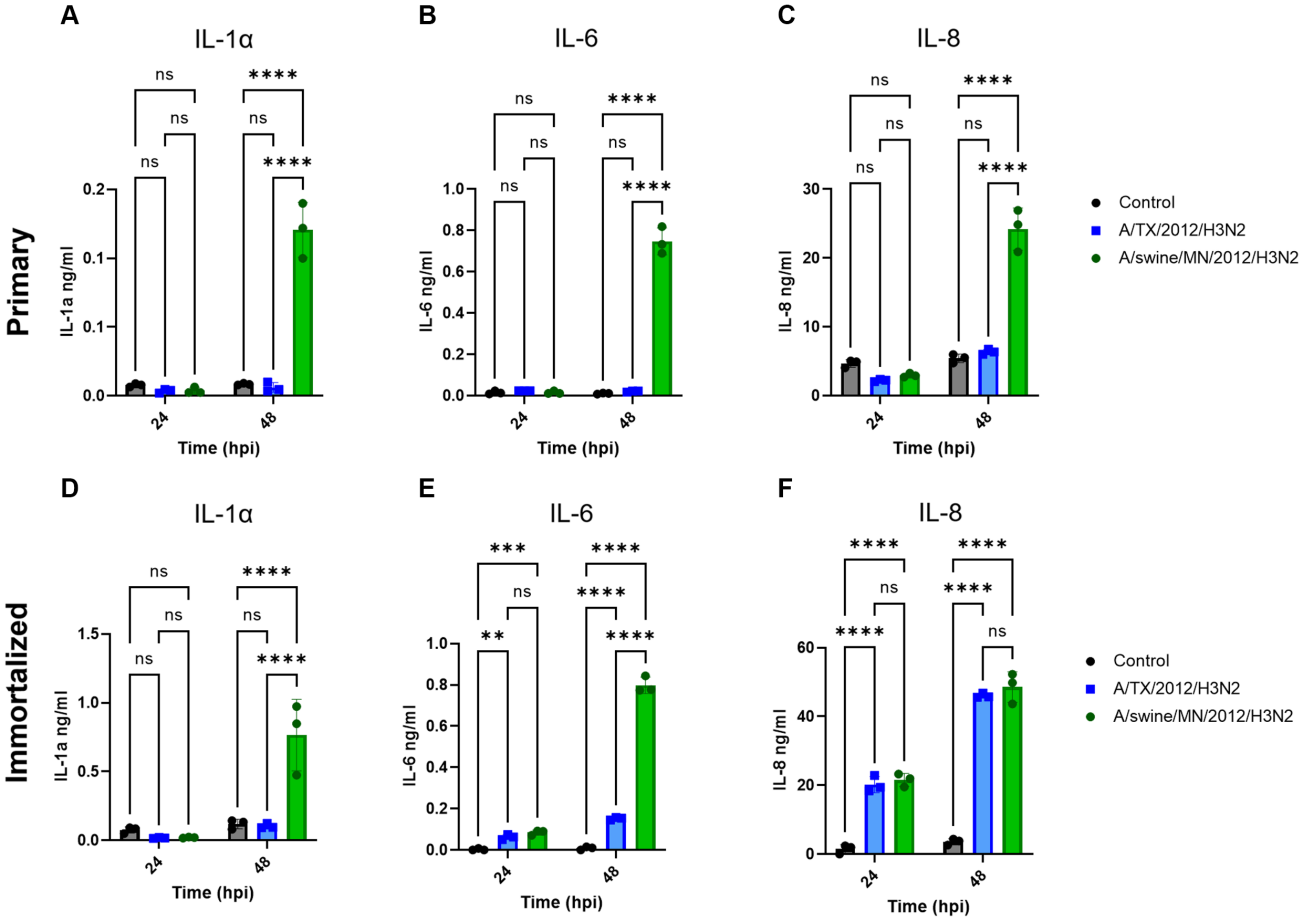
swIAV of H3N2 subtype promotes robust pro-inflammatory cytokine secretion in both immortalized and primary swine respiratory cells. Immortalized (P24) and primary (P6) swine respiratory cells were seeded onto 6-well collagen coated plates and inoculated independently with either control/PBS (black), A/TX/2012/H3N2 (blue), or A/swine/MN/2012/H3N2 (green) at an MOI of 0.1. Supernatant harvested from either primary or immortalized cells at −24 and −48 hpi was tested for **(A,D)** IL-1α, **(B,E)** IL-6, and **(C,F)** IL-8 cytokine/chemokine secretion with MILLIPLEX. Statistical analysis was performed by a two-way ANOVA test. **, *p* < 0.01; ***, *p* = <0.001; ****, *p* < 0.0001; ns, no statistical difference. Error bars represent standard deviation (*n* = 3).

We evaluated IL-1α, IL-6, and IL-8 pro-inflammatory cytokine protein production from the immortalized swine respiratory cells following independent inoculation from either A/TX/2012/H3N2 or A/swine/MN/2012/H3N2. There was no significant difference in production of IL-1α, IL-6, and IL-8 between the A/TX/2012/H3N2 and A/swine/MN/2012/H3N2 infected immortalized swine respiratory cells at 24-hpi (Figures 7D–F). Whilst IL-1α and IL-6 secretion was significantly higher at 48-hpi when infected with A/swine/MN/2012/H3N2 (Figures 7D,E). Additionally, IL-6 and IL-8 secretion was significantly higher at 48-hpi following inoculation from either A/TX/2012/H3N2 or A/swine/MN/2012/H3N2 compared to the control (Figures 7E,F).

## Discussion

The development of an immortalized swine respiratory cell line is a useful tool for the assessment of novel IAV isolates from the field. Swine are an important natural and intermediate reservoir host species and are considered the ‘mixing vessel’ for the reassortment of co-infecting IAV strains in a singular host, leading to the generation of novel strains which may pose a pandemic threat to humans. This is partially due to the highly diverse pool of currently circulating IAV strains in the swine host, which increases the potential combinations of novel strains evolving. In addition, many of the circulating strains incorporate wholly human IAV gene segments into their genome and generate novel strains that possess the molecular tools to transmit and replicate within the human host. The gold-standard *in vitro* model for assessing the pandemic potential of novel isolates at the human/swine interface includes primary respiratory epithelial cells isolated from the host and fully differentiated in ALI. However, given the time taken to fully differentiate the primary cell lines, we were interested in developing an immortalized respiratory epithelial cell line that maintained the characteristic of the primary swine host. This would enable a more efficient pipeline for identifying the transmission and replication success of novel IAV isolates within the swine host and fast-track the development of vaccines against highly virulent strains.

In this study, we harvested and isolated respiratory cells from the bronchi of an 8 weeks-old Yorkshire/Hampshire swine and immortalized the cell line by delivering the SV40-T antigen to the isolated primary cells. We demonstrated that immortalized swine respiratory cell lines were primarily of epithelial origin, as indicated through expression of CK18, which has been used as a marker for respiratory epithelial cells and simple epithelia (23). In addition, staining for potential contaminating cells, such as, fibroblasts and epithelial-mesenchymal transitioning (EMT) cells, revealed that there was a decrease in the percentage of contaminating cells following immortalization via SV40 T antigen transduction (Supplementary Figure S1). However, we were unable to develop a pure population of immortalized swine bronchial epithelial cells and our mixed immortalized swine respiratory cell line contained approximately 30% of undesired fibroblastic or EMT cells.

We demonstrated that both the primary and immortalized swine respiratory cells expressed both α2,3 and α2,6 SA receptors in a similar distribution to what is observed in the lower respiratory tract of the swine host (2, 4, 9). Since, the primary and immortalized swine respiratory cells expressed α2,6 SA, we proposed that both the primary and immortalized cell lines would be permissible to mammalian IAVs and would be an appropriate model to assess strains isolated at the human/swine interface. We measured the replication kinetics of four IAV isolates in both the primary and immortalized swine respiratory cell lines, which included the wholly human-origin A/TX/12/H3N2 huIAV, A/CA/09/H1N1 pandemic strain (pdm09) belonging to the pandemic clade (1A.3.3.2), A/swine/GA/19/H1N2 delta 2 clade (1B.2.1), and A/swine/MN/12/H3N2 swIAVs (Figure 4). The primary swine respiratory cells were permissible to all tested huIAV and swIAVs. However, A/TX/12/H3N2 huIAV replicated to lower titers and for a reduced time in comparison to either of the tested swIAVs, or A/CA/09/H1N1 that originated from the swine host. We hypothesized that this phenomenon observed within the primary swine respiratory cells was due to a lack of adaptation of the huIAV to the swine respiratory cells leading to a robust innate immune response to prevent viral replication. Additionally, we observed similar replication kinetics of the four IAVs in the immortalized swine respiratory cells. Although, the trends observed with the IAV titers were mostly exaggerated in the immortalized swine respiratory cell line, which we theorize could be caused by the immortalization process selecting for a more homogeneous cellular population. In future studies, it would be interesting to further characterize the immortalized cellular population and identify molecular determinants that are selected for during SV40-T antigen immortalization. In addition, to characterize the innate immune responses within sub-populations of immortalized swine respiratory cells that enable enhanced replication of hu- and swIAVs.

We assessed the replication kinetics of an extensive panel of H1N1, H1N2, and H3N2 hu- and swIAVs isolated between 1999 and 2019 in the immortalized swine respiratory cells. All isolates used in the study were permissible in the immortalized swine respiratory cells without the need of trypsin protease to enable IAV entry in the host cell. This implies that protease secretion was not lost following SV40-T antigen immortalization. Mostly, H3N2 huIAVs replicated poorly in the immortalized swine respiratory cells compared to H1N1 or H3N2 strains encoding gene segments of swine-origin. This poor replication of wholly huIAV H3N2 strains has been observed experimentally in the swine host and requires further investigation from both a host and viral standpoint. Surprisingly, we observed similar replication titers and total AUC curve values between pre-pdm09 A/NewCal/99/H1N1, pdm09 A/CA/09/H1N1, and post-pdm09 A/WI/19/H1N1 strains. The pdm strain was a novel reassortment of IAV gene segments of human, classical swine, and avian origin, which was first transmitted to the human host in early 2009. The A/CA/2009/H1N1 strain was able to efficiently transmit between humans, leading to a global pandemic outbreak (24). Accordingly, we were surprised to observe no significant differences between the replication kinetics of A/NewCal/99/H1N1 and A/CA/09/H1N1 in the immortalized swine respiratory cells. However, this observation may highlight limitations in utilizing solely *in vitro* assays to model the complex physiology and immunity of the swine host, which includes multiple physical barriers and cell types that determine the infectivity of IAV within the swine host. Additionally, previous studies have investigated the effects of temperature on the replication kinetics of huIAVs and swIAVs within swine host cells, which we did not address in this study (11, 25). It would be of interest to assess host body temperature and its role as a host factor that IAVs must overcome to efficiently replicate within primary and immortalized swine respiratory cells. In addition, to assess whether temperature influences the replication kinetics of IAVs possessing a specific arrangement of internal gene constellations.

We were interested in measuring the differential pro-inflammatory response of the cell line following inoculation of either hu- or swIAVs. This was because of the differences observed between the replication kinetics of H3N2 subtypes in the immortalized swine respiratory cells (Table 1). In this study, we tested and compared A/TX/12/H3N2 huIAV and A/swine/MN/12/H3N2 swIAV, isolated from the same year and of the same subtype. We found that there was a significant increase in the production of IL-1α and IL-6 following inoculation of A/swine/MN/12/H3N2 at 48 hpi, compared to inoculation from A/TX/12/H3N2, and no significant difference in the secretion of IL-8 at 24 and 48 hpi (Figure 7). Previously, it has been demonstrated that fibroblasts secrete IL-6, and under specific conditions, IL-1α (26, 27). Consequently, it is essential to note that the observed increase in IL-6 and IL-1α following IAV inoculation may have been influenced by the proportion of contaminating cells in the primary and immortalized swine respiratory cell lines developed in this study. Nevertheless, despite this potential influence, we observed a significant increase in IL-1α and IL-6 production following inoculation with the swIAV H3N2 strain compared to the huIAV H3N2 counterpart (Figure 7). In the future, we want to further assess the cytokine production from the immortalized swine respiratory cell line inoculated with a broader panel of hu- and swIAVs. In addition, to extend the assessment of time points to compare the kinetics of secreted cytokines (28, 29).

## Conclusion

We have developed an immortalized swine respiratory cell line that retained most of the characteristics of its primary swine respiratory cell counterpart. The immortalized swine respiratory cell line was still permissive to IAVs at passage 24 and did not require the addition of exogenous trypsin for IAV inoculation, which is mostly observed in primary epithelial cell lines that have undergone airlift in an ALI (9, 30, 31). It is important to continue to identify potential applications for the immortalized swine respiratory cell line for IAV research. In this study, we did not assess whether the isolated primary and immortalized swine respiratory cells could fully differentiate in the ALI system, which warrants investigation because the isolated cell were mostly of epithelial origin. This would provide more flexibility for the researcher to study IAV infectivity in an appropriate model that maintains the biological functionality of the swine hosts lower respiratory tract.

## Data availability statement

The raw data supporting the conclusions of this article will be made available by the authors, without undue reservation.

## Ethics statement

Ethical approval was not required for the studies on humans in accordance with the local legislation and institutional requirements because only commercially available established cell lines were used. The animal study was approved by Auburn University Institutional Animal Care and Use Committee (IACUC). The study was conducted in accordance with the local legislation and institutional requirements.

## Author contributions

PN: Conceptualization, Formal analysis, Investigation, Methodology, Validation, Visualization, Writing – original draft, Writing – review & editing. VP: Investigation, Methodology, Writing – review & editing. JN: Investigation, Methodology, Writing – review & editing. CJ: Investigation, Methodology, Writing – review & editing. MT: Conceptualization, Supervision, Writing – review & editing. CK: Conceptualization, Data curation, Funding acquisition, Investigation, Methodology, Resources, Supervision, Validation, Writing – original draft, Writing – review & editing.
