# Supplementary material for: Development and characterization of an immortalized swine respiratory cell line for influenza A virus research
Source: Front Vet Sci. 2023 Dec 18;10:1258269. doi: 10.3389/fvets.2023.1258269 (PMC10765598; doi:10.3389/fvets.2023.1258269)
Supplement: Supplementary file 1 [file Table_1.docx]

| **Cell Line** | **Passage** | **SV40 T Antigen (Cq)** | **Swine GAPDH (Cq)** |
| --- | --- | --- | --- |
|  |  |  |  |
| Immortalized | P20 | 27.7 | 23.2 |
| Primary | P6 | ND | 24.8 |

**Supplementary Table 1. Immortalized swine respiratory cells were transduced with SV40-T antigen, which was maintained following 20 passages.**

ND = non-detectable.
