# Supplementary figures and images for: Development and characterization of an immortalized swine respiratory cell line for influenza A virus research
Source: Front Vet Sci. 2023 Dec 18;10:1258269. doi: 10.3389/fvets.2023.1258269 (PMC10765598; doi:10.3389/fvets.2023.1258269)

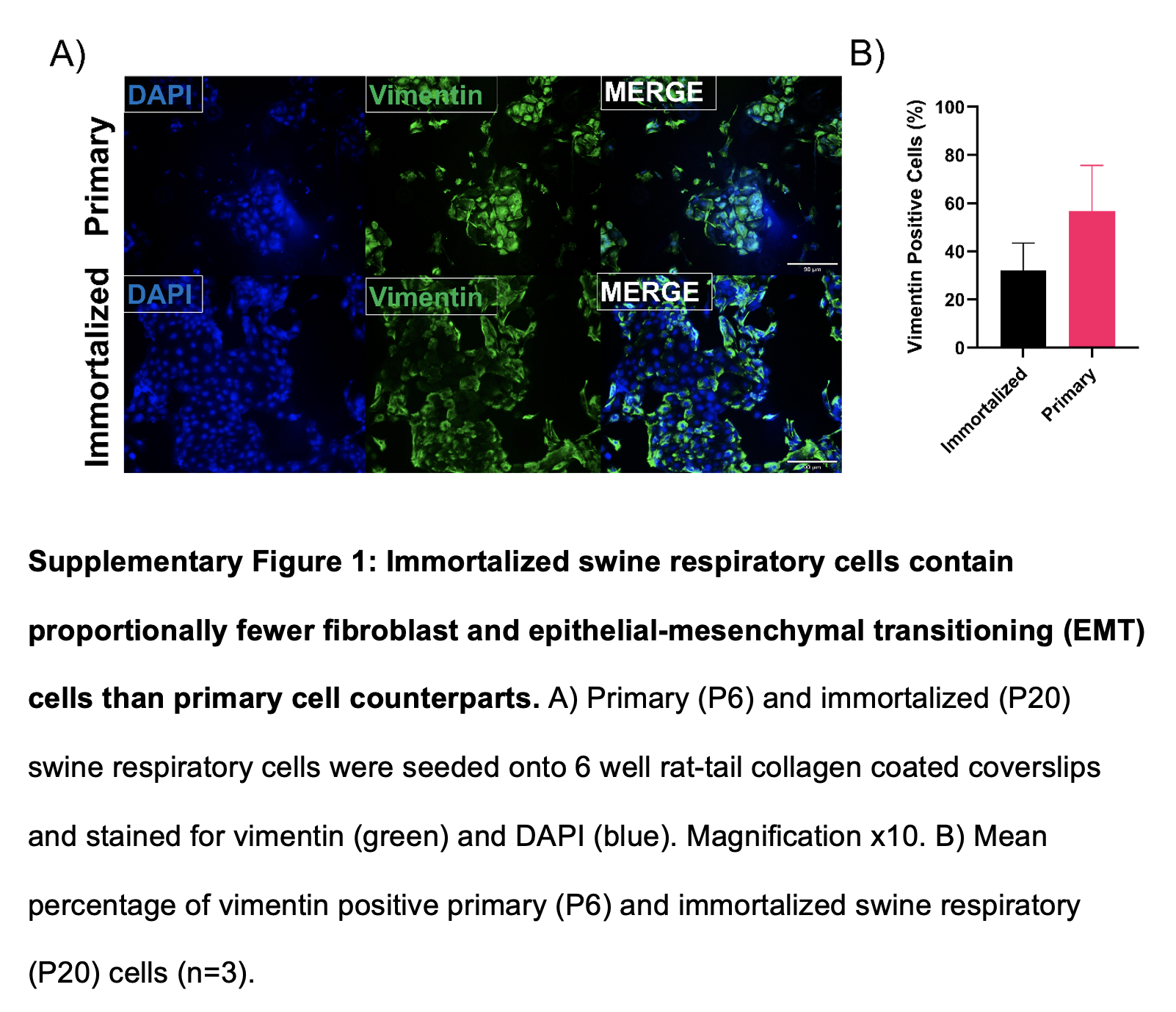

Supplement: Supplementary file 2 [file Image_1.png]
